# Supplementary figures and images for: The Mycobacterium tuberculosis complex pangenome is small and shaped by sub-lineage-specific regions of difference
Source: eLife. 2025 Sep 5;13:RP97870. doi: 10.7554/eLife.97870 (PMC12413193; doi:10.7554/eLife.97870)

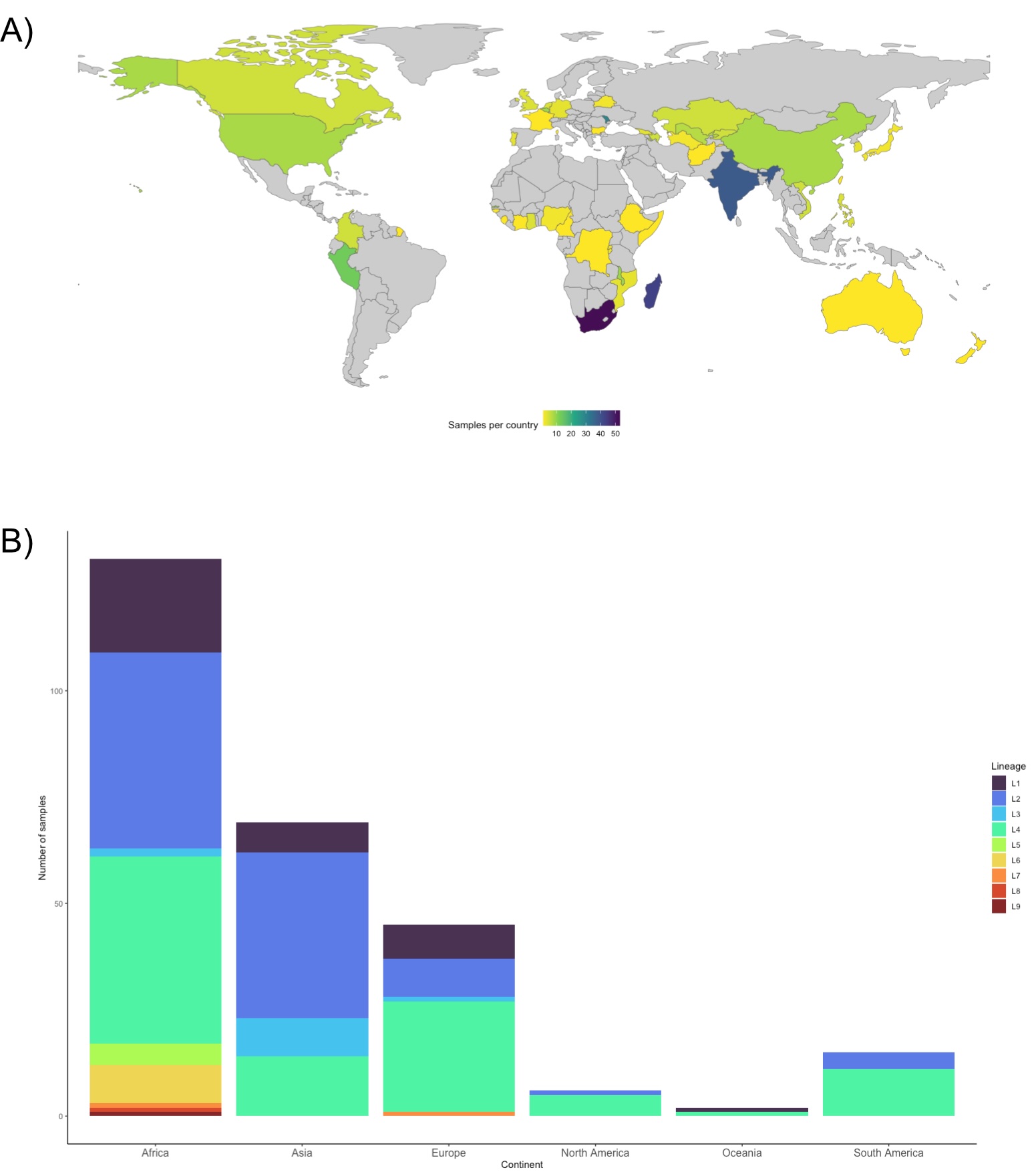

Supplement: Supplementary file 2. — (A) sample collection distribution by country; (B) the number of genomes of each lineage included from each continent. [file elife-97870-supp2.zip › Supplementary File 2.jpg]

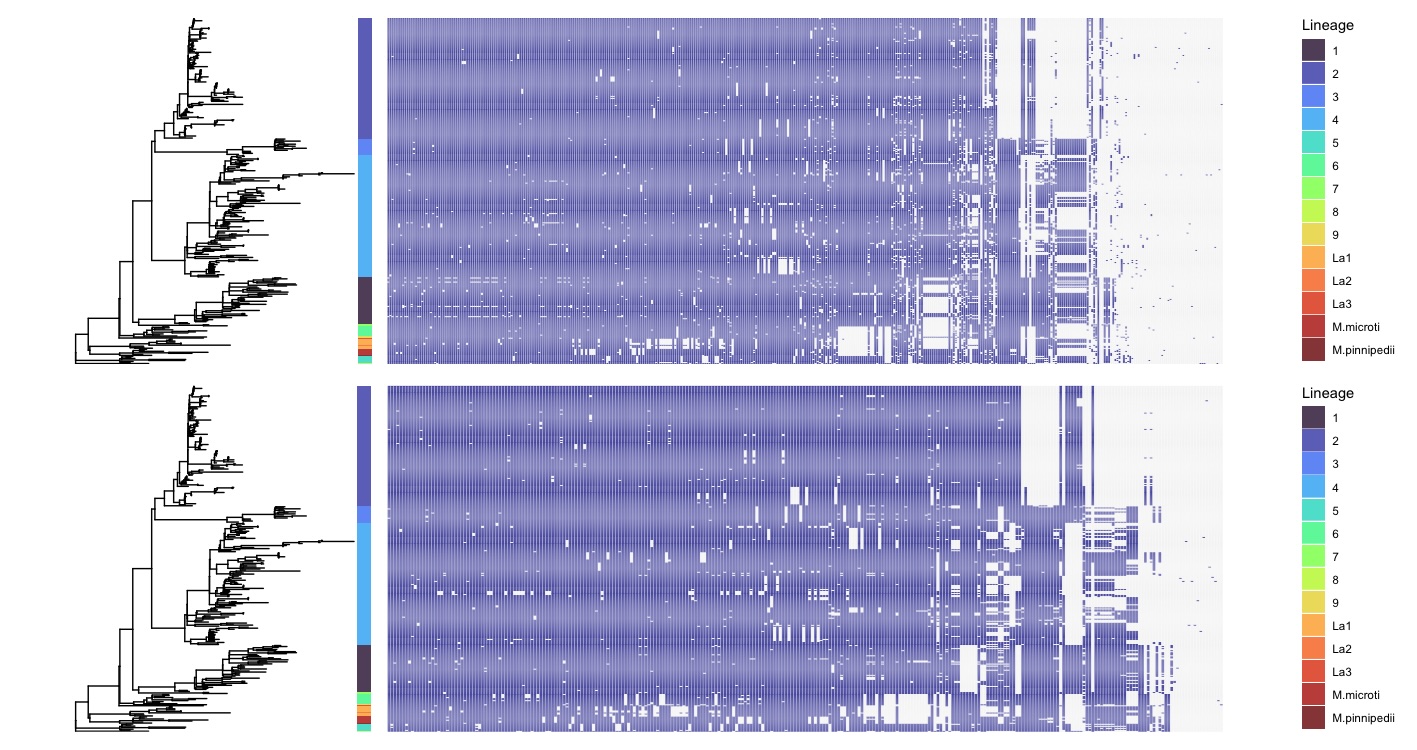

Supplement: Supplementary file 6. — The MTBC phylogenetic tree is shown on the left beside a coloured bar indicating the sub-lineage of each tip genome. The accessory genes/regions are indicated by columns in the heatmap with a blue box if present in that strain’s genome. [file elife-97870-supp6.zip › Supplementary File 6.jpg]

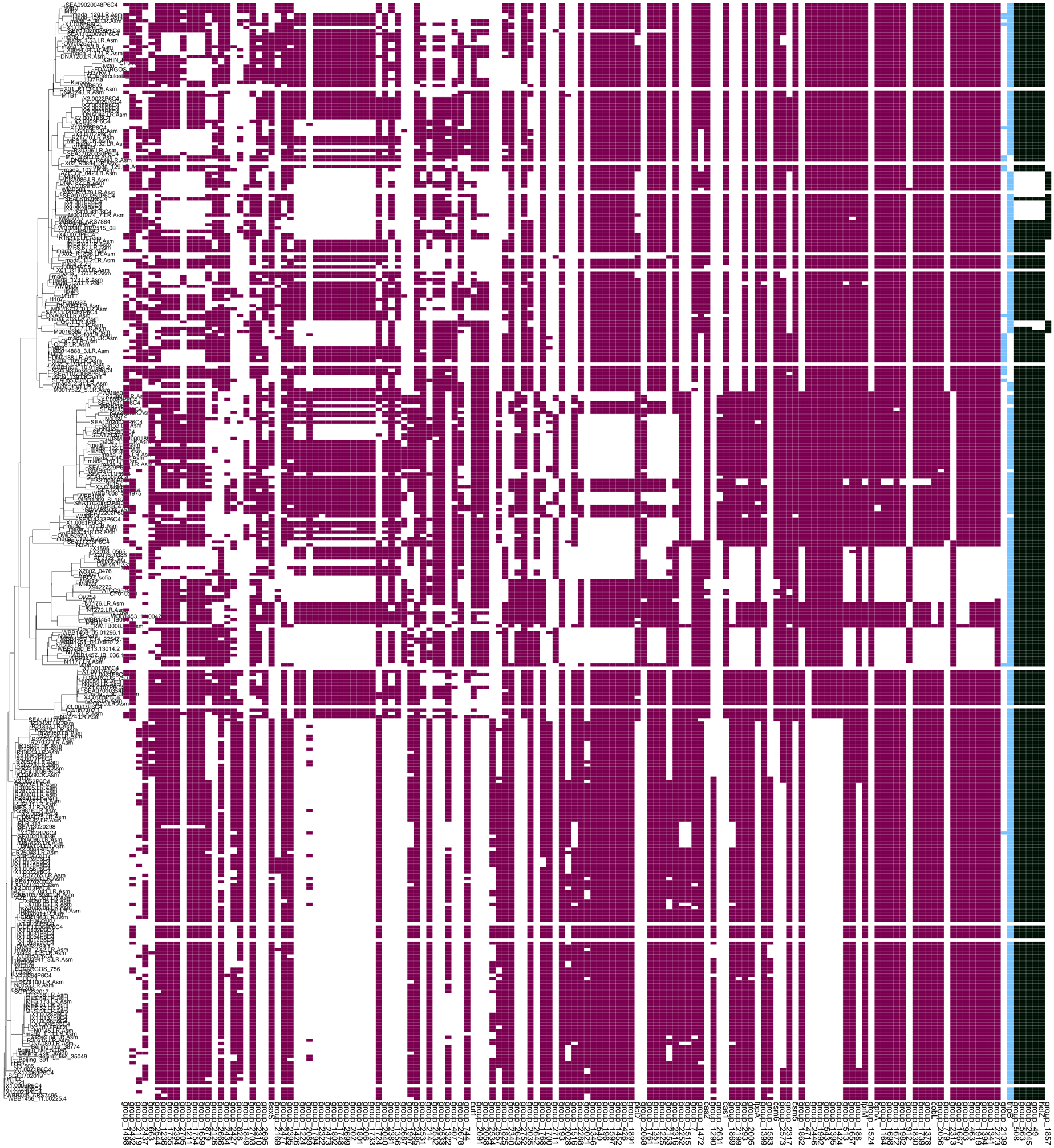

Supplement: Supplementary file 7. — Coinfinder reveals gene association patterns within the accessory genome of the MTBC. Accessory genes, listed on the X-axis, cluster into groups (shown here by varying colours of the blocks). [file elife-97870-supp7.pdf]
