# Supplementary material for: The Mycobacterium tuberculosis complex pangenome is small and shaped by sub-lineage-specific regions of difference
Source: eLife. 2025 Sep 5;13:RP97870. doi: 10.7554/eLife.97870 (PMC12413193; doi:10.7554/eLife.97870)

A) Merged paralogs:

**Fit to Heap's law:**	**Intercept**	**alpha**
	117.6233	2

**Genome Fluidity:**	**Mean**	**Std**
	0.008487286	0.003941261

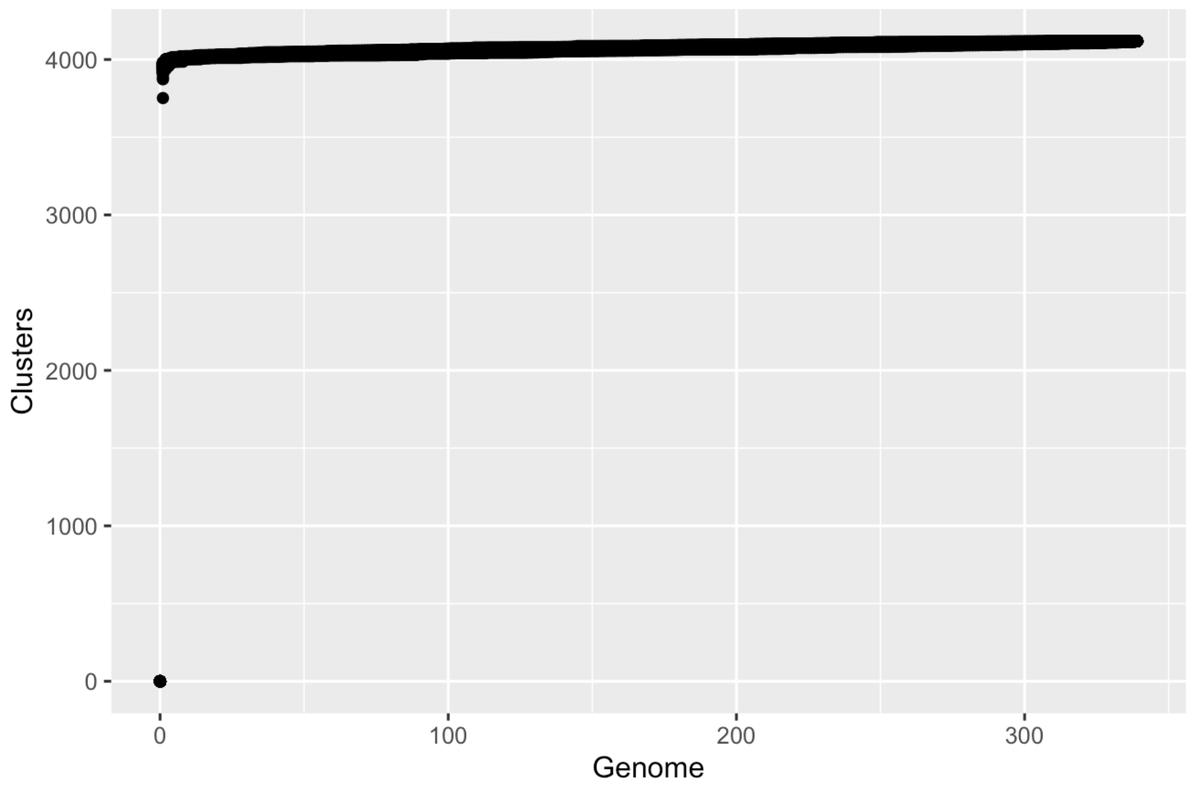

B) Unmerged:

Fit to Heap's law:	Intercept	alpha
	105.10826	1.548288

Genome Fluidity:	Mean	Std
	0.008819889	0.004459309

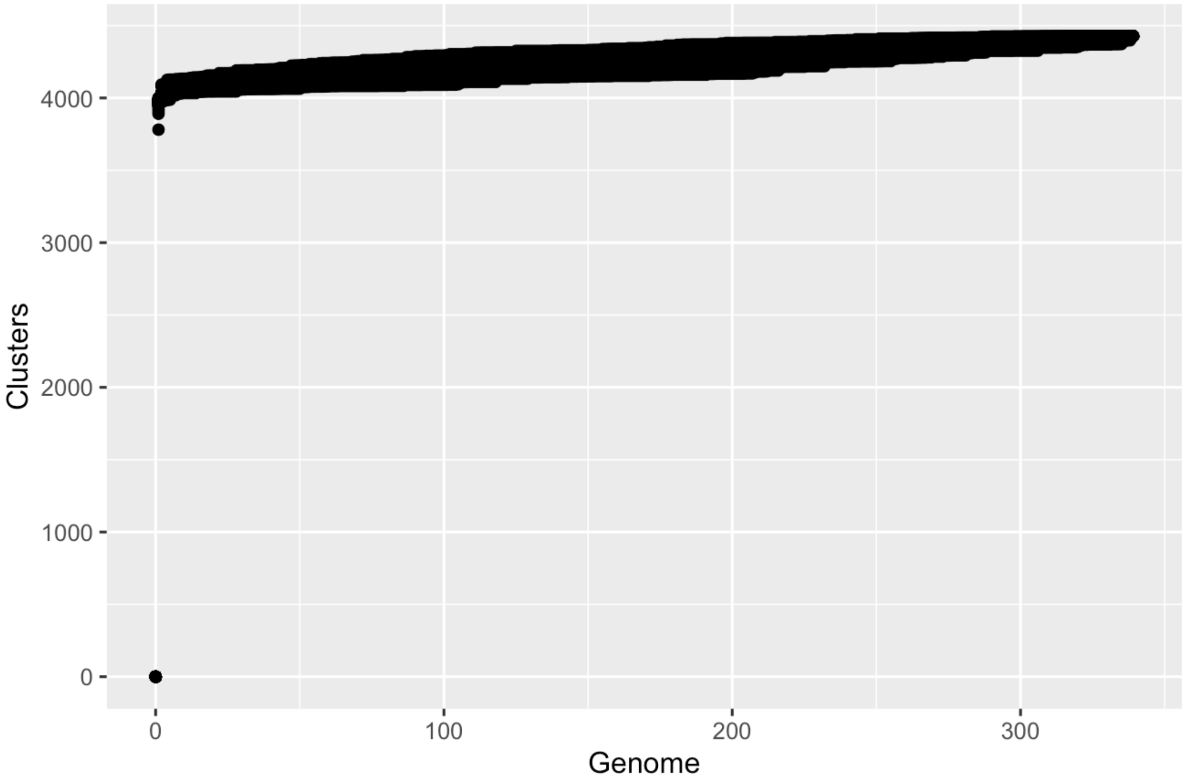

C) Pangraph:

**Fit to Heap's law:**	**Intercept**	**alpha**
	89.29827	2

**Genome Fluidity:**	**Mean**	**Std**
	0.04136684	0.02142143

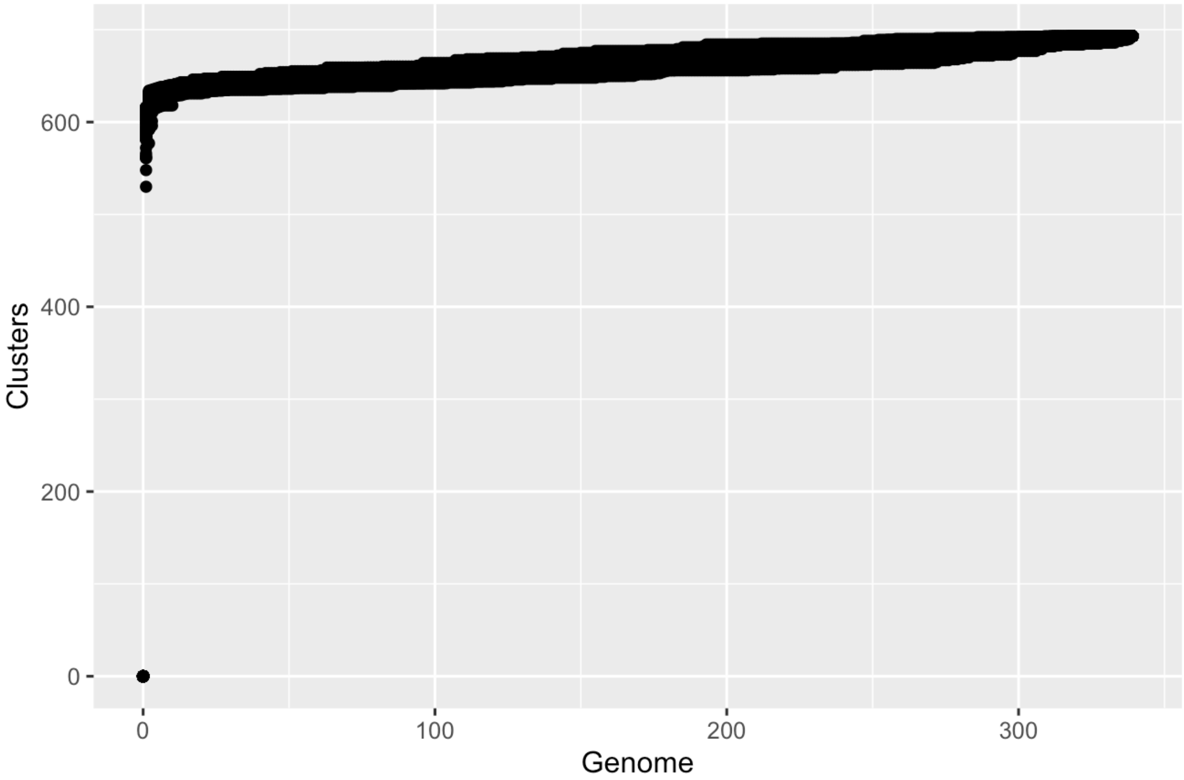

Supplement: Supplementary file 4. [file elife-97870-supp4.pdf]
